# “A Healthy Zebra Not a Sick Horse”: A Cultural Case Study of an Autistic Individual in the USA

**DOI:** 10.1007/s11013-026-10030-3

**Published:** 2026-11-13

**Authors:** Sanehdeep Kaur, M. Ariel Cascio

**Affiliations:** College of Human Medicine, Michigan State University, East Lansing, USA

**Keywords:** Autism, Communication, Double empathy, Biosociality, Collaborative anthropology

## Abstract

Social communication is a key domain at the intersection of culture, medicine, and psychiatry, particularly when communication is medicalized in diagnoses like “autism spectrum disorder” (ASD). Clinicians and scholars debate dominant medical model discourses of autism as well as grassroots neurodiversity perspectives. Increasing influence of neurodiversity perspectives has yielded increasing clinical and communication science studies considering diversity (not deficit) in communication. This cultural case study considers how one autistic young man in the USA orchestrates twenty-first-century discourses about autism, reflecting on his experiences with communication, social disconnect, and sensory sensitivities. His case illustrates how autistic-led concepts such as the “double-empathy problem,” increased attention to sensory-social components of autism, and the changing role of the Internet have substantially reconfigured the voices that autistic adults orchestrate compared with those explicated by previous scholars. These reconfigurations align with changes in medical models of autism that blur and blend with neurodiversity models. His efforts to build supportive, autistic-led spaces provide models for authentic interaction and reveal new tools for forming biosocial autistic community. This cultural case study therefore reinforces the need to avoid one-size-fits-all approaches, and the importance of a person-centered approach to meet the needs of everyone within the community.

## Introduction

### Conceptual Framework

Autism is a salient concept for scholars of culture, medicine, and psychiatry (Cascio, 2018). The “autism concept” (Cascio, 2018) encompasses a particular way of being in the world that was named and described as a specific psychiatric diagnosis in the 1940s in the USA and Austria. In this meaning, autism is characterized by differences in social communication, sensory processing, and repetitive patterns of behavior. It is a diagnostic label, which scholars recognize as entailing both diagnosis as classification (i.e., the naming and categorizing of clinical conditions) and diagnosis as event (i.e., the process of affixing the clinical label to an individual person) (Valtellina, 2018). Scholars also investigate autism as an identity, an object of expertise, an economic fetish around which an autism industrial complex revolves, and more (Cascio, 2018). A rich anthropology of autism has developed over the past decades, with sub-threads including cross-cultural autism studies (e.g., Fein & Rios, 2018; Grinker, 2008), autistic culture studies (Silverman, 2008), linguistics (e.g., Ochs & Solomon, 2010; Sirota, 2010), social movement studies (Cascio, 2020b), and critical autism studies (Woods et al., 2018). This cultural case study of an autistic young adult in the USA is situated primarily within critical autism studies, in that it seeks to avoid reifying autism and rather strives to unpack what autism means in a particular historical and cultural context. It takes a critical disability studies perspective to communication studies as well, asking what different ideas about communication norms, disorders, similarities, and differences are doing throughout the case study. It contributes to the broader anthropology of medicalization, with particular attention to implications for new biopolitical theory (Cascio, 2017) and biosociality (Rabinow, 1999).

This article builds on Bagatell’s (2007) article “Orchestrating Voices,” which presents a case study of Ben, 21 at the time of the study conducted some years before. Bagatell writes about how Ben is constructing his identity in part by navigating different voices around him, in the sense of different discourse about autism. He negotiated between voices of “normalcy” (even a “cure mentality”) encouraging him to fit a certain definition of normal and fit in, and voices of “the Aspie world” offering a sense of “‘normal’ for people with autism.” Bagatell (2007: 422) describes Ben’s identity work as “orchestrating,” namely, “sorting out, juxtaposing, mixing and hybridizing […] the voices or discourses that are part of one’s inner speech” and which come from a variety of sources. As Fein (2020) points out, the music of these voices has changed since 2007. Social studies of autism have highlighted the role of an autism diagnosis, as well as self-identification as autistic, in helping people find that sense of being “a normal autistic person, not an abnormal neurotypical” (Tan, 2018). The diagnosis serves as “biographical illumination” instead of “biographical disruption”; it allows reframing of past experiences and legitimizes alternative communication norms. It is not merely a medical classification, but rather a “reinterpretation of self and identity” (Tan, 2018). Peer interactions allow development of shared vocabularies for bodily and sensory experiences leading to transformation of “bodies with autism” into “autistic bodies”—identity and sensory expression are deeply intertwined in autistic communication (Belek, 2018).

A neurodiversity movement focusing on seeing autism as a difference not inherently a deficit has grown so much that there are now complex conceptual maps unpacking the many different ways social scientists and their participants use the term (Dwyer et al., 2024; McLennan et al., 2025). US youth coming of age on the spectrum navigate different models of autism: medical models pursuing a cure for autism or elimination of autistic traits, neurodiversity models pursuing accommodation and acceptance, and variations within. Fein (2020) traces a twofold model of autism, both as a pathogen (separate from the person) and package (inseparable from the person). She demonstrates how youth are not so much picking one of these models as they are orchestrating the two of them simultaneously. This cultural case study considers what discourses about autism in a young man born roughly a generation after Ben is orchestrating.

### Culture, Medicine, and Communication

Social communication is key to both the diagnosis^1^ and lived experience of autism. Autistic youth in the 2020s must orchestrate voices about culture, medicine, and communication when authoring their self-understanding and creating autistic community. Unpacking this observation sheds light on the cultural meaning of social communication as an object of study. The central role of social communication in the diagnosis and conceptualization of autism shows how autism emerges relationally, i.e., in the interactions between individuals rather than within an individual neurology alone (Belek, 2019; McGuire & Michalko, 2011). In this way, social and medical are not opposing or mutually exclusive domains. The autism concept is one of the areas in which medicine becomes less individualized and more attuned to the social enterprise. While some lines of research—i.e., some voices in the orchestra—have focused on communication traits, differences, and deficits, others have looked at autistic communication as autistic culture (Block, 2015; Davidson, 2008; Silverman, 2008). Some literature has explored autistic–autistic social interactions more broadly than communication, describing experiences such as autistic intersubjectivity (Heasman & Gillespie, 2019), the “certain magic” of autistic–autistic interaction (Watts et al., 2024), and autistic community connectedness (Cage et al., 2022). These perspectives when brought together are an orchestra of different meanings of autism and communication: communication-as-biology and community-as-culture. This orchestration goes beyond a cure-versus-acceptance dichotomy and entails navigating and balancing different ways of understanding social communication. Here, we briefly overview some trends in autism and communication research.

Communication metrics are used as both diagnostic indicators and outcome measures in clinical autism studies. From the 1940s to 1990s, these were primarily deficit-focused studies, which continue today. However, since roughly the 1990s and the increasing influence of neurodiversity perspectives, there have been increasing clinical and communication science studies considering diversity (not deficit) in communication. This changing focus co-occurs with the growing presence of autistic communities, which scholars credit in part to the increasing accessibility of the Internet during this time period allowing autistic people to connect online and asynchronously (Bagatell, 2010; Davidson, 2008).

Countering the deficits framing of the DSM, Milton (2012) coined the term “double-empathy problem” to describe miscommunication between autistic and non-autistic individuals due to mismatched social expectations and cognition styles. This framework has raised research questions investigating if communication difficulties emerged from individual communication styles or contact between different neurotypes, i.e., between autistic and non-autistic people. Blinding studies of autistic dyads, non-autistic dyads, and mixed dyads exhibiting the inherently different communication styles support the argument that mixed-neurotype interaction is less efficient than same-neurotype interactions (Geelhand et al., 2025). Autistic–autistic conversations often exhibit high mutual adaptation and directness, while autistic–non-autistic interactions often exhibit disruptive patterns due to mismatched norms (Heasman & Gillespie, 2019). The communication challenges stem from reciprocal misunderstanding rather than a one-sided autistic “deficit.” Anthropologists further challenge stereotypes that autistic socialization requires predictable structure by ethnographically describing how loosely structured social spaces allow autistic individuals to achieve intersubjectivity without rigid social scripts (Ryan Idriss, 2021).

While some lines of research isolate communication, others, especially since the early 2000s, have increasingly attended to sensory aspects of autism (Russo et al., 2025). Sensory features are included as core components of autism in the DSM-5, under “restricted, repetitive patterns of behavior, interests, or activities” (APA, 2013). However, we recognize that autistic people’s sensory experiences are also social (Clément et al., 2022). Some “sensory-first” clinical models relate sensory to communication (Russo et al., 2025). We consider moreover what Clément and colleagues (2022) call the “layering of the sensory social,” the way that sensory and social experiences cannot be separated from each other. Sensory–social stress can lead to difficulty communicating (Clément et al., 2022; Russo et al., 2025). All these aspects are tied up together and play their part in the orchestra of voices about autistic communication, community, and culture.

New biopolitical theory (Cascio, 2017), particularly biosociality, suggests one way to look at this interaction. The “bio” in biosociality alludes to views of communication-as-biology and the “sociality” to communication-as-culture. Biosociality refers to how people form individual and collective identities based around a notion of shared biology, in this case that autistic people see themselves as biologically similar to each other and form social, advocacy, and political groups around that sense of shared similarity (Buchman et al., 2011; Friend, 2014). Biosociality has been a common way of looking at autistic sociality, though it has its limits (Cascio, 2017; Ryan Idriss, 2021). Cascio has questioned its applicability in Italian offline settings, speculating that the theory may have limited applicability outside the USA or outside the affordances of digital spaces (Cascio, 2017, 2020a). Anthropologists working in USA have also argued that offline autistic-led spaces rely on autistic people’s intentional, and sometimes exhausting, social labor, rather than biosociality, to build the social infrastructure of their community (Ryan Idriss, 2021). Below, we unpack how one autistic young adult in the USA conceptualized biology, sociality, and possibly biosociality.

This report investigates the differences in communication styles between autistic and non-autistic individuals by exploring lived experiences of an autistic person. It highlights not only the varying communication styles but also sheds lights on stigma, coping mechanisms, and sensory challenges faced by autistic individuals. We argue that the autistic-led theory building of concepts such as the “double-empathy problem,” increased attention to sensory–social components of autism, and the changing role of the Internet in social life have substantially reconfigured the voices that autistic adults in the USA are orchestrating, and that these voices provide new tools for forming biosocial autistic community groups.

### Research Design

This case lies at the intersection of person-centered ethnography and collaborative anthropology. Person-centered ethnography (Hollan, 1997; LeVine, 1982) investigates cultural phenomena—in this case, the intersection of culture, medicine, and psychiatry in communication—through an in-depth exploration of an individual’s experience. While ethnographic and anthropological studies involve many forms of collaboration, collaborative anthropology “*deliberately* and *explicitly* emphasizes collaboration” (Lassiter, 2005, emphasis in original). Collaborative approaches such as participatory and community-based anthropology aim to reduce power imbalances in research (Schensul et al., 2014). This approach also aligns with disability studies’ call for “nothing about us without us” (Charlton, 1998) and the specific framework of participatory autism research (Fletcher-Watson et al., 2019). Scholarship at similar intersections (e.g., Frank, 2000; Morton & Thomas-Smith [A.K.A. Mr. Black], 2009) has fruitfully contributed to the study of culture, medicine, and psychiatry by unpacking the impact of context in an individual’s life, especially the lives of socially marginalized individuals. We use these approaches to emphasize the participant’s unique experiences and perspective of cultural and social contexts.

### Data Collection

Neither of the authors identify as autistic. Cascio is an anthropologist with over ten years of experience working with autistic interlocutors. The participant himself contacted Cascio and expressed his desire to contribute his experience as a case study that would showcase his experiences as an autistic individual from his own perspective. Kaur, a medical student roughly the participant’s age, conducted in-depth, semistructured interviews via Zoom, sometimes with Cascio present, and an initial in-person meeting to provide the participant a platform to share his personal narrative. This initial meeting was the first time Kaur met the participant or specifically sought dialogue with an autistic person. Despite being steeped in neurotypical communication norms, Kaur found rapport building and dialogue easygoing. Reflection on these norms informed the dialogue (e.g., when the participant commented on his own conversational style during the conversation) and the authors’ interpretation (e.g., grappling with the conflicting ideas about autistic communication, noting our own surprise or lack of surprise comparing these data to the literature, our previous experiences, and our awareness of cultural discourses about autism).

During these interviews, the participant provided insights into his communication style, social interactions, and everyday challenges. The initial in-person conversation when the participant was 24 years old was entirely participant-led and unstructured. The later online conversations were organized around a semistructured interview guide. The interview questions regarding the diagnosis were divided into three parts: before the diagnosis, the diagnosis, and after the diagnosis to explore how his self-perception and understanding of his social experiences evolved over time. Kaur typed responses on a document shared on screen, and the participant offered comments and clarifications at the time and afterward. His feedback during writing focused primarily on (1) small factual matters including details and specific words; (2) adding further context to included stories that foreground the meaning he makes of them and would convey; and (3) providing more direct quotes to explain concepts in his own words, including feeling the need to change himself, components taking up mental energy, and the role of occupational therapy in his story. He has affirmed he was satisfied with the contents of this report.

### Ethics

The Michigan State University Institutional Review Board (IRB) determined that this case study did not constitute human subjects research under federal regulations. In keeping with common case study practices at our institution, we created a formal consent form outlining the participant’s permission to include individual credentials, right to withdraw from the publication at any time before press, and the right to review final report prior to publication. We discussed process and consent throughout our conversations, and the participant signed his agreement to the initially submitted manuscript after an opportunity to review it and provide feedback.

The participant, Henry Fellows Moss, specifically requested to include his real name in the report. He wanted to take ownership of his experiences and being identified was an important aspect of sharing his story. This report dwells on the close collaboration with him and, rather than treating him as a research subject, serves as a platform for his voice and perspective. This person-centered strategy reflects an approach where the participant actively shapes how his narrative is shared. Henry did not wish to be an author.

### Case Study

Henry Fellows Moss is a 24-year-old male diagnosed with autism spectrum disorder (ASD). He completed his Bachelors in General Studies from the University of Michigan. He was initially diagnosed with Asperger’s syndrome as a child, which is now categorized as part of ASD in the DSM-5. In addition to autism, he has other co-occurring diagnoses such as attention-deficit/hyperactivity disorder (ADHD)-inattentive type, generalized anxiety disorder (GAD), and obsessive–compulsive disorder (OCD).

He was first diagnosed with Asperger’s syndrome at the age of 3 but “[doesn’t] know what led [his] parents to get [him] looked at.” Ever since he was young, he had a “strong particularity” toward things being in a certain way. A story his parents told him is that as a child, he would run around the couch in a specific way, and then, the person sitting on the couch would have to “touch [his] hand in a particular way” and if they wouldn’t, he “was furious.” “When mom told [him] about [his diagnosis of] Asperger’s syndrome at 14, [he] did not agree with the diagnosis at the time” until he “investigated in college.” Once he got into college, he started reading more about autism and ended up with what he calls his “self-diagnosis” of ASD in summer 2022, based on the ASD criteria in DSM-5.^2^ When asked if he had met with a psychiatrist to get an official diagnosis under the new criteria, he explained that in 2023, he told [the psychiatrist that he] was autistic” and the psychiatrist “simply notated it” without any additional questions. This distinction between learning of his diagnosis and his later self-diagnosis highlights the complex ways autism operates as a diagnosis and as an identity; Henry agentively orchestrates different sources of information from different time periods to come to his current self-understanding.

Prior to the self-diagnosis, Henry describes feeling the need to change himself. He explains:

I thought that there was an objectively right way to socialize and an objectively right way to interact with others and that I was supposed to learn this and get better and better at doing it. And that my first impulses directing me to speak or move or position my body or make facial expressions were simply faulty. And that I had, by observing the interactions of others, to train myself to imitate them. I didn’t realize that I had a set of instincts that equipped me to interact tremendously well with other autistic people without conscious effort of training myself to some standard. That there was a set of people among whom my very first impulses were correct, and would lead to harmonious interactions.

He felt that his “first impulses” were often “in conflict with what [he] thought was some objective standard of proper socialization.” During late elementary school, he thought of autism being “pathological” and as “something that made people defective and incompetent.” Growing up, he would “conceal signs of autism” which included things from “how [he] hold[s his] body to the way [his] facial expressions work in response to others, to words that [he] use[s], the subjects [he] brings up.” In these ways, Henry explicitly engages with the voices of “normalcy” Bagatell describes with Ben.

When making his self-diagnosis in 2022, Henry “felt somewhat protected” from doubts due to a former diagnosis of Asperger’s syndrome during childhood. Self-diagnosis provided “a sense of relief” and made him feel like “a healthy zebra not a sick horse.” This comment alludes to the catchphrase often taught in medical education *“When you hear hoofbeats, think horses, not zebras.”* This phrase aims to remind physicians and trainees to consider the most obvious explanations first. Therefore, communities such as rare disease advocates and neurodivergent advocates have taken up “zebra” as a self-description and symbol for their experience (Hunter, 1996). Henry’s rephrasing describes both relief and validation that autistic people are not “sick horses” with something wrong, but “healthy zebras.” Self-diagnosis provided reassurance that there was not something inherently wrong with him. Rather, he “was wired to communicate with other autistic people and that where [his] social instincts were misaligned with those of broader society, they were well aligned with those of autistic people.” Henry is orchestrating alternative understandings of autism that challenge deficit-based interpretations.

After confirming his childhood diagnosis through his own research and self-diagnosis, the way Henry perceives himself has changed especially “when [he is] in groups of autistic people—[he is] quite graceful and charismatic.” This grace contrasted with having to conceal signs of autism and practicing how to communicate with others. When in the company of other autistic people, he explains he “could act on [his] first instinct in a very genuine and non-self-conscious way.” He regards his autistic social skills as “excellent” as he feels at ease when communicating with an autistic person as his “first instincts in communication are usually right and do not require so much review and revision before being acted upon.” In contrast to the voices of “normalcy” Henry describes engaging prior to his self-diagnosis, this perspective brings in an alternative voice, evoking something like the double-empathy problem, focusing on relational components of autistic communication and specifically the ease of being in autistic groups.

Resources such as the personal insights and perspectives of other autistic people, along with writings by other autistic people on online forums, also served as voices in this orchestration process, contributing to a deeper understanding of the diagnosis. One of the aspects of being autistic that he finds valuable is the “directness and forthrightness and honesty” in communication. These resources led to the realization that:

it really does seem that autistic people are wired to communicate specifically with each other and that through communication with other autistic people we can understand that our instincts for communication are not wrong and that we are not broken or malformed. The experience of having your first instincts at communication go well is very affirming in that way.

Moreover, Henry noted that he has a higher resistance to conforming to societal pressures than a non-autistic individual.

In contrast to these social skills in autistic-autistic interactions, interacting with non-autistic people takes Henry a great deal of “mental energy” and feels “inauthentic” when he has to “stifle certain autistic traits.” This feeling happens when he is constantly monitoring, questioning, and denying his autistic instincts—“not behaving in a way that comes naturally to [him].” If comfortable with a non-autistic person, he makes them aware that if he is “fidgeting, it doesn’t mean that [he’s] bored or anxious.” Other repetitive behaviors that he finds himself repeating include rocking back and forth, pacing when on the phone, nodding when stationary, and “prefer[ing] to move around” because that makes him more comfortable. Monitoring and masking these bodily components is only one part of what takes up this mental energy. The other components relate to monitoring and revising “[his] impulses as to what to say and when so that they are more in line with neurotypical expectations” so as not to “alienate or annoy the other person,” rather than “spontaneously saying what comes to mind as [he] can with other autistic people.” He has to constantly monitor his own and other’s speech for subtext—intended or unintended—and respond accordingly “in a way that [he does] not have to with other autistic people.” These nuances of mental energy management highlight the inseparability of social communication and sensory considerations, with sensory understandings of autism being another important voice in Henry’s narrative.

Indeed, Henry finds that the world has not been built to suit the needs of an autistic person. One example of this is that sensory challenges, specifically the loudness and chaos, “often prevent [him] from going to restaurants or dancing” and is “extremely distressing.” He has trouble eating outside at restaurants as the “car noise” is bothersome. Now, he is “a lot more gentle” with himself and is “much more careful about attending to [his] sensory needs rather than ignoring them.”

Sensory needs are the main area in which Henry has sought help from professionals. He worked with an occupational therapist for a couple months. As he explains:

I reckoned that there were likely areas in which I was not meeting my needs in which I did not know that I could meet them. There were probably sensory improvements that could be made that it simply had not occurred to me to make because of a lifetime of ignoring my own sensory needs. And that by doing this with someone trained to do precisely this, I could move the needle significantly forward on my own wellbeing.

While Henry did not seek any academic accommodations for autism, he did receive them for ADHD. He also mentioned that he has a “fear [of] telling providers” about being autistic because he feels “doctors may take me less seriously.” He “want[s his] medical concerns taken seriously,” and does not “want to lose any sort of autonomy just because [he’s] autistic.”

These experiences motivated Henry to start a non-profit organization—The Delian Club^3^ for autistic adults. It is a space for autistic individuals to “simply show up” to “alleviate the loneliness that many of us experience.” The club provides a way to drop the constant self-monitoring and to be truly oneself when in company of other autistic individuals which helps you “come to understand that there is nothing wrong with you and that you are not less skilled at interacting.” Henry personally finds “online interactions very unfulfilling” and prefers to have in-person conversations. While he uses online tools like email and Zoom to schedule and arrange details, the Delian Club is explicitly focused on in-person events.

## Discussion

Being aware of an autism diagnosis can substantially shape one’s interactions in a social context. For some, it can provide relief and provide justification for how their interactions were the way that they were, alluding to “biographical illumination” (Tan, 2018). On the other hand, for some, it can cause alarm resulting in a heightened sense of self-monitoring and constant evaluation of how others might perceive them. Diagnosis can be affirming and enable autistic people to embrace the autistic communication norms, while others might experience increased scrutiny and social pressure. This section will discuss Henry’s experiences as an autistic individual in relation to existing literature, with prevailing themes of autistic-autistic communication success, social disconnect, sensory sensitivities, and in-person autistic gatherings.

During the interview, Henry emphasized that the communication challenges between autistic and non-autistic individuals is mutual rather than one-sided. This perspective aligns with the “double-empathy problem” as operationalized in some empirical research suggesting that autistic adults communicate better and were more comfortable with another autistic person and vice versa in the case of non-autistics (Jones et al., 2024). Autistic communication skills are often misunderstood by non-autistic people, even though autistic people can build strong connections when their communication style is accepted and matched. Controlled research suggests that autistic individuals exhibit different verbal behaviors primarily in mixed-neurotype interactions.  Communication was more fluid within the autistic dyads and non-autistic dyads (Geelhand et al., 2025) and autistic people have effective communication skills when communicating among each other as compared to when the communication is between autistic and non-autistic groups (Crompton et al., 2020). Autistic-autistic interactions are often experienced as more natural, validating, and less effortful compared to interactions with non-autistic individuals. These interactions lead to improved emotional well-being, a stronger sense of belonging, and reduced self-stigma (Watts et al., 2024). Similarly, Henry describes feeling more comfortable when communicating with other autistic individuals which echoes how such interactions contribute to greater social ease and overall quality of life.

The way Henry describes autistic-autistic communication orchestrates different ideas about social communication. His description of “first instincts” in communication aligns with a communication-as-biology understanding of autistic communication as innately “wired” without being deterministic. Communication-as-culture is important too, as he describes experiences in autistic groups as affirming for developing a non-deficit understanding, and providing the circumstances in which to be graceful, charismatic, and genuine. In this telling, shared biology—being “wired to communicate specifically with each other”—prompts social groupings characterized by particular communication styles—e.g., “directness and forthrightness and honesty”—that in turn prompt a sense of ease. He frames misunderstandings in non-autistic spaces not as failure of communication on his part but as mismatch between different communication expectations. This further supports how Henry orchestrates biological, cultural, and interactional narratives to make sense of his experiences.

In non-affirming contexts, these divergent communication styles can lead to heightened anxiety among autistic individuals, especially in high-stakes environments such as healthcare settings. Misinterpretation of autistic communication remains common in both clinical and public settings, contributing to systemic barriers. Communication mismatches not only result in interpersonal challenges, but also poor healthcare outcomes and broader societal exclusion referred to as a “triple empathy problem” (Shaw et al., 2024). Henry echoed this concern when he expressed a fear that his autonomy would not be respected if his healthcare providers knew about his diagnosis. When the implicit expectations of typical body language, i.e., nonverbal communication are not met, it results in misinterpretation among autistic people and healthcare providers regardless of one’s actual communicative abilities (Strömberg et al., 2022). Moreover, autistic adults who self-reported “identified needs were significantly less likely to be met in a work environment than in a home environment” (Bradshaw et al., 2023). In high-stakes settings such as legal or evaluative interviews, certain behaviors in autistic adults may be misinterpreted, underlining the need for informed assessment approaches to avoid biased judgments (Logos et al., 2025).

Socialization is another aspect that is closely tied to communication and can also be understood in a difference-not-deficit framework. Difficulty with (neurotypical) social interaction potentially contributes to lower rates of (neurotypical) social participation, friendships, or close relationships (Chan et al., 2023). Henry started the Delian Club to provide autistic individuals with a platform for in-person socialization among the autistic community. Indeed, there is literature finding that autistic-led peer spaces foster peer communication contexts. These spaces are often rooted in identity affirmation where norms align and reduce the communicative strain as well as promote more authentic social engagement (Heasman & Gillespie, 2019; Tan, 2018; Watts et al., 2024). Autistic–autistic dialogues feature high levels of mutual adjustment and directness, which contributes to smoother interaction and shared understanding (Heasman & Gillespie, 2019). Such communication flourishes in flexible, low-pressure environments developed by autistic people themselves, where social participation is voluntary, expectations are relaxed, and intersubjectivity emerges organically (Ryan Idriss, 2021).

Sensory challenges are a prominent aspect of autistic adulthood that significantly impact daily functioning and quality of life. Many autistic individuals experience heightened sensitivity to auditory and tactile stimuli leading to sensory overload in environments that others may find tolerable. These sensory needs are not uniform and can vary widely, creating barriers to communication and avoidance behaviors resulting in increased anxiety (Strömberg et al., 2022). Interactions at routine social environments such as workplaces, restaurants, or public transport can be overwhelming due to sensory stimuli (Penton et al., 2023). Henry describes how loud noises make it difficult for him to enjoy the dining experience at restaurants and being in social spaces with other sensory stimuli. His experiences illustrate how sensory hypersensitivity can restrict participation and increase psychological stress contributing to social isolation. Henry’s narrative also shows the inseparability of sensory–social layers (Clément et al., 2022). Just as sensory stress can inhibit social communication (Clément et al., 2022), reduced sensory stress and shared sensory experiences can promote it. These sensory experiences provide a common topic for autistic people to discuss together, further strengthening the in-group understanding and mutual support (Belek, 2018).

Online communication has become more prominent over the last few decades, allowing autistic culture to flourish (Bagatell, 2010; Davidson, 2008). It serves as a platform for autistic individuals to connect, express themselves, and build social networks in a more accessible and less sensory-intensive environment (Nguyen et al., 2020). This environment reduces stress, allows more control, and avoids the constant worry about confusing social cues (Hassrick et al., 2021). Despite the potential benefits reported above, Henry prefers face-to-face interaction over interacting online. He perceives online interactions as “unfulfilling” and lacking the emotional depth and authenticity he experiences during direct interactions. Online interaction does not always reduce loneliness or replace in-person friendships, and some struggle to form meaningful connections (Hassrick et al., 2021). While there are certainly vibrant autistic cultures online (Egner, 2022; Lupton & Southerton, 2024) and offline (Ryan Idriss, 2021), Henry’s experiences reinforce the heterogeneity of these cultures, the need to avoid one-size-fits-all approaches, and the importance of a person-centered approach to meet the needs of everyone within the community.

While this cultural case study sheds light on these processes, it remains a single story, with the inherent limitations to generalization that come from any single story (Adichie, 2009). We do not claim that Henry’s experience is representative of autistic individuals in the USA, rather that it is illustrative of some of the cultural context that autistic individuals navigate. Our collaborative approach to this case study, with an identified yet non-authoring participant, has precedent but remains somewhat innovative. To address potential limitations of clarity with respect to our different roles in collaboration and interpretation, we have provided information on our positionalities and Henry’s feedback. We hope these details can be useful both for critical autism studies and for methodology scholarship.

## Conclusion

Henry’s story highlights the voices autistic young adults are orchestrating in the USA in the 2020s. Henry engages with deficit and difference narratives around communication, social interaction, and sensory sensitivities. His narrative highlights the indivisibility of sensory–social domains in lived experience. Henry’s personal experiences online and efforts to create in-person social opportunities through the Delian Club highlight the impact of the changing role of the Internet as a tool for, rather than alternative to, in-person social interaction.

Henry’s experiences also reflect changes in the social landscape of autism since Bagatell’s discussion of orchestrating voices. While Bagatell emphasizes autistic identity construction, we focus on communication, community, and culture as aspects of being autistic that are key to this identity construction. In Henry’s case, the range of available voices have substantially expanded. He not only draws upon clinical concepts but also neurodiversity-oriented perspectives, autistic community narratives, interactions within autistic social spaces, and his own lived experiences of communication and belonging. The growing neurodiversity movement, autistic-led advocacy groups, and expanding autistic-autistic communication opportunities (both online and in person) have created new avenues for community formation and identity development. Henry’s participation in autistic social spaces provide not only social connection but also resources for understanding oneself and one’s place within the broader autistic community, including autistic-led theories and concepts such as the double-empathy problem and increased attention to sensory experiences. He does not simply adopt a single explanation for autism but actively orchestrates these voices into a coherent account of himself as an autistic person.

While recognizing the need to move beyond new biopolitical theory in accounting for autistic sociality (Ryan Idriss, 2021), and the explanatory limits of these approaches (Cascio, 2017, 2020a), we do find many aspects of biosociality and biological citizenship that resonate with Henry’s story. Henry describes a biosocial similarity to other autistic people, a sense of not only shared identity but also shared sensory–social experiences and communication styles. Orchestrating these meanings has provided Henry tools for his own life and for creating community with autistic peers. Notably, he orchestrates these voices in a context in which autistic and non-autistic people disagree over concepts of autism, communication, community, and culture. His story therefore highlights for scholars of culture, medicine, and psychiatry the fluidity of these concepts between and beyond clinical and non-clinical dichotomies.

## Data Availability

No datasets were generated or analyzed during the current study.

## Footnotes

1 The *Diagnostic and Statistical Manual of Mental Disorders—fifth edition* (DSM-5) (American Psychiatric Association [APA], 2013), dominant in the USA, frames autistic communication differences as deficits. This viewpoint has been challenged by emerging research in which autistic communication is considered distinct, not deficient (Cummins et al., 2020). The *DSM-5* maintains atypical communication and social behaviors as the key indicators of autism, which has prompted multifaceted research into autistic social interactions (Senouci et al., 2021).

2 These criteria include persistent deficits in social communication and social interaction; restricted repetitive patters of behaviors, interests, or activities; the presence of symptoms in the early developmental period; impairment in social, occupational, or other functioning areas of life; and inexplicable only by intellectual disability or global developmental delay (APA 2013).

3 The name of the club is a reference to the ancient Greek god Apollo, sometimes called the Delian.
